# Fire system safety risk cognition model and evaluation of major public safety risks

**DOI:** 10.3389/fpubh.2022.987277

**Published:** 2022-09-02

**Authors:** Wen Li, Xuesong Lu, Xu Dong

**Affiliations:** School of Architectural Engineering, Huanggang Normal University, Huanggang, China

**Keywords:** system security risk, risk characteristics, real risk cognition, evaluation model, fire

## Abstract

Along with the expansion of city scale, and the increase in the density of population and buildings, the occurrence of a major public fire safety incident in cities will lead to a serious threat to the overall public safety and the sustainable economic and social development of the relevant region. A simple and practical safety risk assessment method of fire prevention in gas stations is of great value for disaster prevention and application in key industries. The constituent elements of a gas station fire prevention safety system are complex, and include equipment, materials, environment, operation, and other factors. This element of information has randomness and temporal dynamics. To promote the transformation of the safety supervision mechanism of gas stations, realize the dual objectives of risk classification and risk dynamic management, and control the gas stations' safety systems the gas stations safety systems are the objectives of our paper. By taking the “fire” risk point of fire prevention in gas stations' system as the research object, this paper puts forward the cognitive path of fire prevention in gas stations' safety system for risk disasters, and explains the coordination between characteristics of inherent, initial, and real risks and the structure of the risk system's attributes. A realistic risk assessment model of fire risk with inherent and dynamic risks is established. An example was introduced to apply the real risk model, and the results were consistent with the actual prediction results, thereby showing the effectiveness and practicability of this method. This risk assessment method can provide a scientific basis for the prevention of fires and control of the fire prevention safety system, showing the changes in risk levels in different stages, and providing risk warning for project managers in taking prompt corresponding risk control countermeasures and improving the efficiency of risk management.

## Introduction

Fire is the most frequent and destructive disaster in urban gas stations. Most of these accidents are caused by the corrosion of pipeline equipment (1), inflammable materials, and dense personnel at the gas stations, causing large casualties and property losses. With the increase in supply and demand of gas stations, the causes of accidents are diversified, and there are widespread posts, links, and operations pertaining to out-of-control pipe leakage. Therefore, prevention of these major risks requires advanced control, and must highlight risk prevention and control of key equipment, technology, materials, places, and other system attributes to employ effective means of ensuring urban fire safety. As such, it is key to evaluate whether prevention of major risks is efficient to strengthen the control of labor-intensive sites and high-risk processes, and reasonably define the key parts to be controlled such as hazardous substances, processes, equipment, sites, and operations. Therefore, it is of great practical significance to conduct an in-depth study on the assessment of fire risk at gas stations and discover assessment indicators that are more consistent with the actual situation of the city, establishing a more reasonable assessment system.

Risk has many different definitions (2–4), and often referred to as the combination of probability and consequences of a specific hazardous event. Most of the early risk matrix methods used the description method or scoring method to determine two grades to measure the degree of risk: the qualitative scale of possibility and risk severity (5, 6). This discrete scale limits its application in evaluation (7). Garvey et al. (8) and Qazi et al. (9) focus on traditional risk identification technology that emphasizes the necessity of system interaction between capturing risks. Qazi et al. (10) believed that decision-makers encountered risks at all stages of risk management, and proposed that the risk matrix method could effectively deal with risk dependence and risk bias. Although traditional risk analysis methods have been widely used, such as formal safety assessment, event tree, fault tree, and analytic hierarchy process that have been used for risk analysis and prevention, there remain some defects, such as insufficient quantification of risk estimation (11). To resolve the above shortcomings, some advanced evaluation methods were proposed, such as Bayesian networks (12, 13), evidential reasoning, and the fuzzy logic reasoning model (14–18). Although it is beneficial to improve the integrity of data, it fails to analyze the causal relationship between risk data. These models are only applicable to risk assessment at a site or risk point, but not that of security systems.

Through the investigation of major accidents, it is found that the causes of accidents are often accompanied by new unknown risks, meaning there is a scenario gap; risks are accompanied by dynamic uncertainty (19), the unexpected nature of natural disasters (20), new technologies (21), and other scenarios that constantly emerge, and their risk consequences are dynamic. In other words, due to the uncertainty of risk, its state is dynamic and accompanied by diversified industrial accident incentives, and the widespread existence of out-of-control management positions, links, and regions, also makes decision-making based on risk assessment results uncertain (22–24). In actual management, there is a gap between known risks and unknown risks, which leads to inconsistency in the expected accident scenario. Only by compensating for this defect and investigating the causal relationship between risk changes and the occurrence of major accidents can we effectively prevent them.

Since the operation process is a dynamic continuous process, to reflect the changing trend of risk accumulation, most scholars consider system security risk as the main line and propose new research methods. Hou et al. (25) discussed and analyzed the framework of hazardous chemical leakage accidents from two aspects of the safety management system and emergency rescue. Merch et al. (26) pointed out that most of the available risk assessments and occupational health and safety were originally designed to meet the needs of large enterprises, and it seems to be widely accepted that the overall accident rate would be significantly higher when applied to small enterprises, resulting in a gap. Lindhout et al. (27) proposed that there is a significant gap between the major accident scenarios predicted by the company's safety management department and the actual situation. This finding points out that there is a gap in the results of the risk assessment method, and led to proposing a major risk assessment method for systematically dealing with “unknown risks,” concluding that making comprehensive preparations for hazard identification and risk assessment in safety management was impossible. Lindhout and Reniers (28–30) analyzed potential hazard scenarios and proposed effective measures to mitigate these potential hazards through the set scenarios and a series of management modes related to industrial processes. Zhang et al. (31) rebuilt the framework of accident prevention in the construction industry, identified the main causes of construction accidents as a whole system, and then decomposed them into 6 sub-systems, 16 hierarchical factors, and 39 sub-factors. This paper defines the construction accident causation system model by using accident causation theory and a system thinking method. Gjerdrum and Peter (32) pointed out that due to the uncertainty of “risk preference,” the risk list may be incomplete, meaning that there are many factors of “unknown risk,” proving once again that risk factors cannot be fully identified. Existing system assessment methods do not fully consider online monitoring of risk assessment and new risks arising from changes in technology, products, operations, and organizational structure in industrial development (33). The system risk assessment method has strong applicability. Although the above research has achieved significant results in major risk identification, risk structure mode, and assessment method of specific scenarios of system risk, it is also proposed to study the prevention of major risks in key industries considering many risk factors. However, in practice, there may be risks in operation due to unexpected changes in equipment or substances, deterioration of process equipment, and failure of online monitoring (34, 35). Given the problem that risk identification in complex systems is not hierarchical and focused enough, and the dynamic correlation of causality is ignored, there will be a large deviation between the risk identification results and the actual situation. To highlight the key prevention and control parts of safety system risk identification, and improve the completeness and accuracy of the identification process, it is necessary to reconstruct the system safety risk assessment model and apply it to key industries under control.

According to the new requirements of standards and regulations related to risk assessment methods of the global industrial development, risk management pays increasing attention to simple and widely applicable methods, and tends to focus on researching for comprehensive risk assessment methods with high-risk positions and online dynamic monitoring. For example, Xu and Chen (34) proposed a theoretical framework for systematic risk assessment based on equipment and facilities, materials or energy, environment, and operation in dangerous industries, and formed a major risk index system of key industry safety systems, including inherent risk index, risk control index and dynamic risk index. Based on this theoretical system framework, Li et al. (35) established a quantitative method for assessment of an inherent risk of system attributes, considering the disturbance caused by the management state to inherent risk, proposing a correction method of inherent risk, and forming the realization risk assessment model. These studies provide a good theoretical framework, but the systematic quantitative evaluation needs further research.

To sum up, an increasing number of scholars have adopted different analysis methods based on different assessment perspectives for related research on risk identification, index analysis, and assessment model. Although they provide certain theoretical and technical support for risk management, prevention and control, most scholars' risk assessment is based on research on humans, property, and management. Lack of hierarchical and feedback analysis on the inherent risk of the system, the effectiveness of the management level, and the influence of external environmental disturbance index factors. In addition, most of the previous studies focused on risk identification analysis in the internal or local system operation stage of enterprises, and lacked systematic analysis and research on attributes and management status indicators of fire safety management systems, as well as a structural description of the construction of these indicator systems. Consequently, the risk of the fire safety system based on the analysis of the cognitive and system security attribute index makes it necessary to explore a suitable set for fire local static, a dynamic and complex system as a whole system for the dynamic control theory of risk identification and evaluation methods, pay attention to the transfer between elements, a dynamic feedback effect, make the safety risk identification and the evaluation of dynamic research appear more valuable for application. This provides a reference for realizing the online risk dynamic control platform of the public fire prevention and safety system.

## System security risk reconstruction and assessment process

A new approach to system risk regards system security risk as a whole assessment system. Starting from the risk source, a study on the inherent attribute risks of equipment, facilities, materials or energy places, processes, and high-risk parts of the operation is performed, and is then combined with the initial risks formed after the protection of existing safety protection measures, and finally the dynamic risk impact after the emergence of new information is considered. Consequently, a series of risks are finally manifested as real risks. Therefore, the system risk assessment model would be reconstructed before studying the real risk. To prevent major risks from being pre-controlled in advance, highlighting and strengthening the key control of labor-intensive places and high-risk processes, and reasonably defining the key control parts such as equipment and facilities, materials or energy, places, processes, and operations are key to evaluate whether the prevention of major risks is efficient.

### Analysis of system security risk characteristics

Most studies ignore the inherent properties of system risk, that is, the uncertainty and dynamic change information in specific scenes (36). Chen et al. (37) established a risk grading assessment method for enterprises in chemical industrial parks based on the source of inherent risks, effective prevention mechanism, and vulnerability of receptors according to the risk system theory and characteristics of chemicals, but the influence factors of dynamic change are weakened. Considering the inherent risk perspective as the main line, the inherent risk of system attribute and the dynamic risk related to the complex system security risk were analyzed again. To understand system security risks, it is necessary to clarify the main relationship between system elements, to facilitate the analysis of the transformation of system risk events in different dimensions. The concepts of system security risk are defined as follows:

Inherent risk refers to the energy (e.g., electric energy, potential energy, mechanical energy) inherent in equipment and facilities, materials or energy, etc. The hazard source exists objectively, and its attribute determines that the accident will cause serious consequences, once it happens. Thus, inherent risks are considered risks that do not take into account existing control measures. Therefore, the inherent risk is objective, and the fire prevention system risk refers to the risk of harmful equipment and facilities, materials or energy, operation, and others.

The formation of initial risk can be regarded as the inherent risk of system attributes and the uncertainty of local system management state triggered together. It is the risk present after considering the existing safety protection measures based on the inherent risk. In other words, the degree of risk depends not only on the degree of inherent risk, but also on the level of prevention and control, and the environmental sensitivity of the recipient (37). Therefore, based on the inherent risks of system attributes, the frequency index generated in the process of management and control is introduced to modify the inherent risks, and thus the initial risks can be obtained. The relationship is expressed in Equation 1:


(1) $$\begin{array}{l} R_{0}=H\times G \end{array}$$

where *R*_0_ is the initial risk, *H* is an inherent risk, and *G* is the risk of key controlled objects.

The uncertainty of risk involves whether the risk and the subsequent consequences after the risk will occur. Risk is an objective existence independent of people's consciousness and not subject to people's will. In other words, we can only measure the risk generated by a certain event and take relevant measures to avoid or reduce the risk, but we cannot prevent the occurrence of the risk. However, risk has certain controllability. Under certain conditions, the risk can be judged by a series of measures and certain means can be used to avoid or reduce the risk. In addition to randomness, there is a certain inevitability between risk and probability. The inevitability of risk in its frequency is that risky events must have consequences. Therefore, the operation process is a dynamic continuous process, which creates real risks.

Actual risk refers to a system under actual control by external disturbances, and not subjected to subjective control of a dynamic state that may lead to the occurrence of accidents. That is, the aggregation of initial risk and dynamic risk constitutes a real risk. Paying attention to the actual risk assessment of the system and the transmission and dynamic feedback between risk elements has more practical value for the research of safety risk identification and dynamic assessment of complex systems.

The actual risk is represented by *R*, and its value is calculated by integrating the initial risk *R*_0_ with the dynamic risk correction coefficient *K*_*m*_. The actual risk can be expressed as a function in Equation 2:


(2) $$\begin{array}{l} R=R_{0}\times K_{m} \end{array}$$

In the face of a complex safety system, the risk management model of coordinating major risk system attributes and dynamic regulation is explored from the inherent risk, initial risk, and real risk of the system, and the internal, external, and internal logical relationship between different factors in the occurrence process of risk events is revealed. General risks can be further discussed when a mature risk management system integrates a big data and other technologies to facilitate data acquisition in the future. Given this situation, focusing on the background of major risk events in the system, combined with the actual production situation of the enterprise in the safety management work, tracking and tracking, explores the coordination mode between system attribute risk and dynamic risk structure elements, which is helpful for clarifying the coordination mode between system risk elements, risk structure, and evaluation objects.

**Figure 1:**
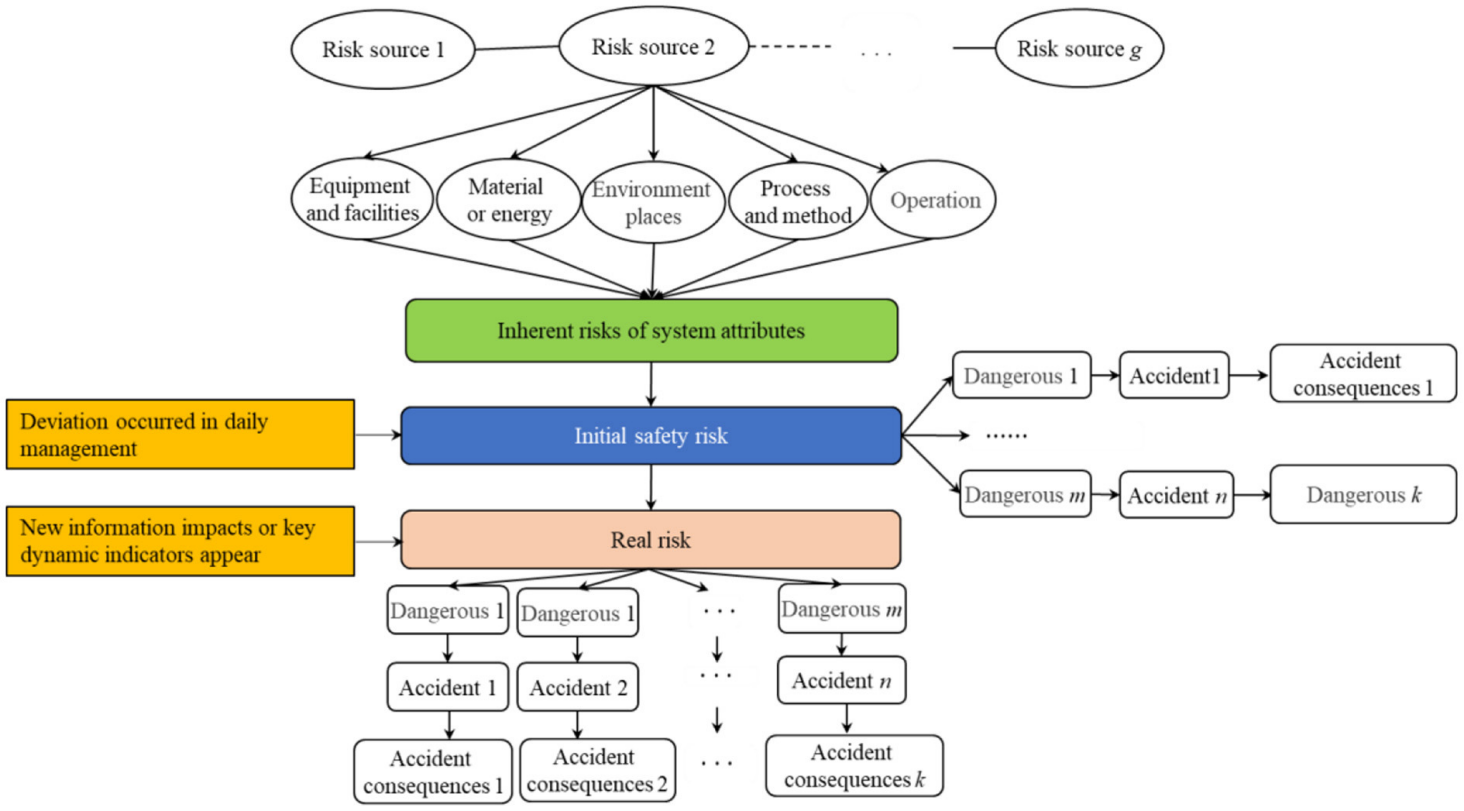
Main logic line of risk structure.

Different from the traditional simple system risk assessment method, the system has complex constituent elements and many evaluation elements. Hence, it is necessary to adopt the key prevention and control method, that is, a “dimension reduction” idea. Based on the importance of the system attribute prevention and control object, the research focus is to adapt its risk prevention and control ability. To reflect the changing trend of risk accumulation, build a logic line of risk change as shown in Figure 1, and explore the changing trend of major risk system attributes coordinated with dynamic regulation from the inherent risk, initial risk, and real risk of the system.

### System security risk assessment process

**Figure 2:**
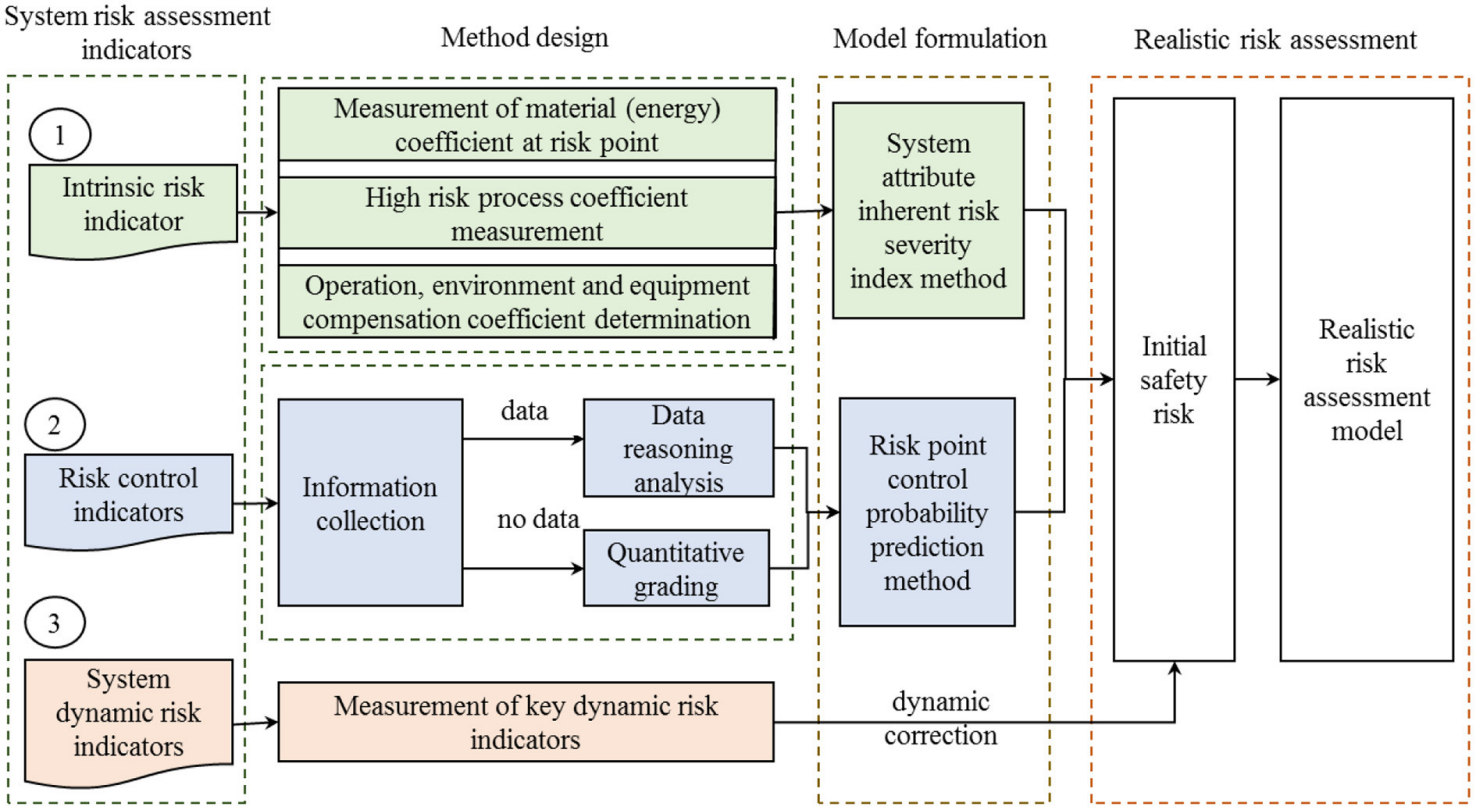
System risk assessment process.

System security risk includes static risk and the dynamic risk of the whole system. The former represents the objective static characteristics of the system and the control changes of local risk management status. The latter reflects the dynamic situation of the system as a whole caused by external influences, such as online monitoring, early warning feedback results, continuous rainstorm, earthquakes, special periods, and other factors affecting the overall risk status of the system with timely changes. Although the emphasis of the two analyses is different, there is a certain correlation. From the perspective of management, the dynamic risk assessment of the system as a whole is convenient for managers to clarify the dynamic risk of the whole system, while the dynamic risk analysis of static risk and local management is convenient for managers to arrange targeted risk control measures in daily life. Only by cooperating with them can the risk assessment achieve the best practical effect. However, most scholars focus on risk analysis of some common accidents or local system problems, ignoring the coordination between dynamic risk assessment of the system as a whole, static risk, and local dynamic risk analysis. Next, the system attributes are quantitatively analyzed to form a risk point initial risk and unit initial risk classification model, which can reflect the causal logic relationship of accident risk points. Then, according to the input dynamic hidden danger data, special period data, natural disaster data, and other evidence of the online monitoring system, the timely and dynamic risk assessment of the fire prevention system attribute is carried out to provide a theoretical basis for managers. Therefore, the key dynamic indicators are used to modify the system attribute indicators, and the dynamic risk indicators are used to adjust the risks in each unit of the fire prevention system. In other words, the initial risks of the unit are modified timely to form the real risks in line with the actual state of the system. Combined with system attribute indicators and key dynamic risk indicators, a realistic risk assessment model is constructed, as shown in Figure 2.

Firstly, as shown in the system risk assessment process in Figure 2, a quantitative method of “five key parameters” is proposed for the equipment and facilities, material or energy, place, process, and operation compensation coefficient of the inherent attributes of the risk point to quantify the severity of the inherent risk index of the fire prevention system. Secondly, to solve the difficult and static problems of obtaining the risk analysis data of disturbance factors, the initial evaluation parameters are obtained by taking the risk control indicators as the center. Then, the inherent risk severity index and risk control frequency index are aggregated into the initial risk of the risk point. Finally, according to the characteristic values of monitoring items, the key dynamic risk index correction method is proposed, and the initial risk is dynamically corrected. After the dynamic timely correction, the real risk assessment model is integrated to form the timely risk assessment coupled with system attributes and management status.

## Methodology

**Figure 3:**
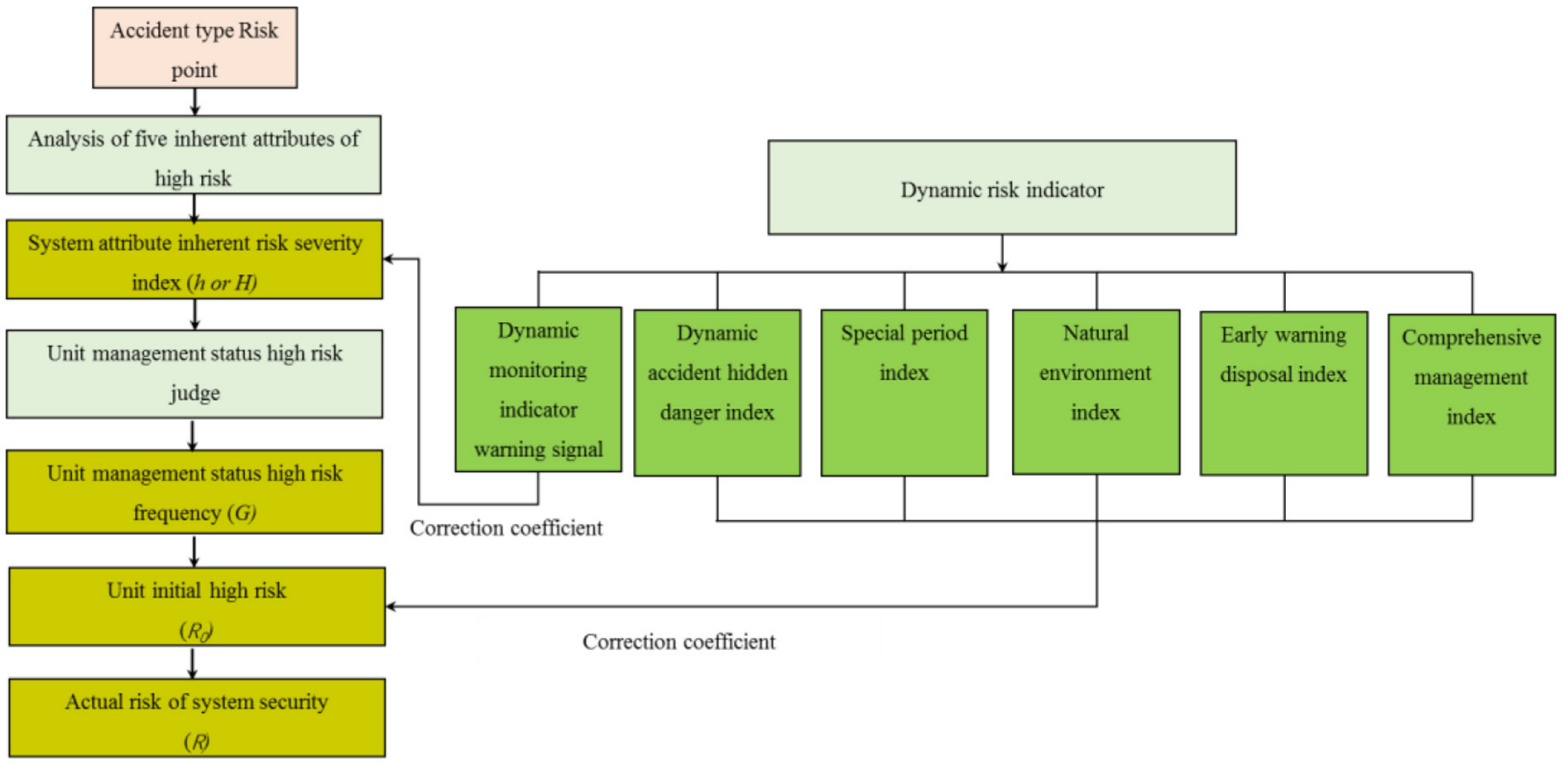
Realistic risk assessment model.

Our study aims to prevent and defuse major risks and fire risk point system attributes and management status of timely risk assessment as the background. Thus, a realistic risk assessment model is constructed, as shown in Figure 3, that is different from traditional risk assessment methods, based on the systematic risk cognitive structure model, analysis system property risk point of equipment, materials, place, process, operation inherent risk indexes and quantitative methods, to manage state and local controls risk frequency calculation method, considering the key dynamic risk indicators of overall risk.

### Determination of inherent risk index of fire risk point

The places or regions that may induce serious accidents within the unit are considered risk points, and the fire risk points are often highlighted in the gas station fire prevention system. The inherent risk of system safety is classified according to the five major disaster-causing factors of major accidents in order to solve the inherent risk, and the steps are as follows:

**Step 1:** Hazardous equipment and facilities are measured by the intrinsic safety level of storage tanks at risk points.

**Step 2:** Hazardous substances are determined through the energy characteristics of the substances in the storage tank at this risk point.

**Step 3:** A dangerous place refers to the exposure risk index of personnel within the risk point.

**Step 4:** A dangerous process refers to the failure rate of static eliminators, alarm devices, monitoring videos, and other monitoring facilities.

**Step 5:** Dangerous operations refer to high-risk operations that are involved, such as special operations, dangerous operations, special equipment operations, etc.

The inherent risk index *h* calculation method of risk points is shown in Equation 3:


(3) $$\begin{array}{l} h=h_{s}\cdot M\cdot E\cdot K_{1}K_{2} \end{array}$$

where, *h*_*s*_ is an index of dangerous equipment and facilities, ranging from 1 to 1.7; *M* is the dangerous material or energy risk coefficient, ranging from 1 to 9; *E* is the exposure index of personnel in dangerous places, ranging from 1 to 9; *K*_1_ is the correction coefficient of monitoring failure rate, with *K*_1_ = 1+*p, p* being the average value of monitoring facility failure rate; *K*_2_ is the risk correction coefficient of dangerous operation, with *K*_2_ = 1+0.05*q, q* being the number of high-risk operation types involved in risk points.

### Unit high-risk frequency index calculation

An inherent risk mainly emphasizes the inherent risk of the objective existence of substances, and the quality of management only represents the high and low probabilities of risk events. The greater the accident probability, the greater the risk control intensity, indicating that the gas station safety system risk events are more likely to occur. However, the risk of an inherent risk will not disappear, but the change of the management state in the process of risk management will make the inherent risk fluctuate, so the complement of the occurrence probability of uncontrolled management is used as the risk control to measure the fluctuation of inherent risk. Unit high-risk management frequency index is expressed as Equation 4:


(4) $$\begin{array}{l} G=1+P \end{array}$$

where *P* is the accident risk point event probability.

### Dynamic risk correction

The dynamic risk indicator regulation aims to correct the initial risk by taking the real risk status as the parameter. It mainly refers to the real-time correction of early warning results of online monitoring characteristic indexes. Other indicators contain five key dynamic risk factors including dynamic accident hidden danger data, the impact of holidays and other special periods, the impact of natural disasters, and the impact of governance measures to timely modify the initial risk of the unit.

(1) The inherent risk severity index (*h*) of risk points was modified by using the warning signal coefficient *Km*, a characteristic index of online risk monitoring. The real-time alarm of online monitoring items is divided into a level I (low alarm), a level II (medium alarm), and a level III (high alarm) alarms. When the online monitoring items reach three level I alarms, it is recorded as one level II alarm; when the monitoring items reach two level II alarms, it is recorded as one level III alarm. Given this, after several rounds of tests, the base number of the corresponding dynamic monitoring characteristic index coefficient *Km* is set to 1.20, and the dynamic risk correction expression of the online monitoring characteristic index's early warning signal is established (Equation 5):


(5) $$\begin{array}{l} K_{m}=1.20\left(\text{a}+2\text{b}+3\text{c}\right) \end{array}$$

where *a* is the number of yellow warnings, *a* = 0, 1, 2, and 3; *b* is the number of orange warnings, *b* = 0,1, and 2; *c* is the number of red alerts, *c* = 0 or 1.

(2) Other dynamic indicators to modify the initial high-risk security risks of the unit

i) Dynamic accident hidden danger index correction According to the accident hidden danger data reported by the enterprise, if there are only general accident hidden dangers, but no major hidden dangers, the initial risk (*R*_0_) of the unit shall be modified according to the number of hidden dangers based on the accident investigation standard 100: If the number of hidden dangers is between 1 and 5, *k*_*i*_ =1.20; if the number of hidden dangers is between 6 and 20, *k*_*i*_ =1.40; if the number of hidden dangers exceeds 20 times, the correction rules of major accident hidden dangers shall be implemented. As long as there is a major potential accident, the dynamic indicator correction coefficient *k*_*i*_ is directly used to correct the initial unit realistic risk (*R*0). The value of the dynamic indicator regulation coefficient *k*_*i*_ is shown in Table 1.

**Table 1:** Values of dynamic risk index correction coefficient.

**Initial risk (*R*_0_)**	**Correction coefficient** ***k_i_***			**Correction coefficient**
	***i* = 1**	***i* = 2**	***i* = 3**	***kc***
I level	1.20	1.44	1.73	0.84
II level	1.30	1.69	2.20	0.67
III level	1.40	1.96	2.74	0.50
IV level	1.50	2.25	3.38	/

Here, i represents the adjustment times of other dynamic indicators, and direct adjustment of more than 3 times is considered a major risk.

ii) Special period index correction Special periods refer to statutory holidays and important national or local activities. In the quantification of indicators during special periods, the initial unit realistic risk (*R*_0_) is corrected by the dynamic index correction coefficient *k*_*i*_. The value of *k*_*i*_ is shown in Table 1.

iii) Correction of natural environment index For the quantification of natural environment indicators, such as earthquakes, and debris flow, the dynamic index correction coefficient *k*_*i*_ is used to correct the initial unit actual risk (*R*_0_). The value of *k*_*i*_ is shown in Table 1.

iv) Correction of early warning disposal index If a yellow warning information appears, then the enterprise failed to dispose within 24*h*. In the case of an orange warning information, then the enterprise did not dispose within 12*h*. The dynamic index correction coefficient *k*_*i*_ is used to correct the initial unit actual risk (*R*_0_). The value of *k*_*i*_ is shown in Table 1.

v) Comprehensive management index revision If the enterprise adopts the comprehensive management of the closed and sold gas stations, the system risk will be significantly reduced. Therefore, the correction coefficient *k*_*c*_ is adopted to reduce the initial unit realistic risk (*R*_0_), as shown in Table 1.

### Actual risk of unit

(1) Dynamic correction of unit inherent hazard index As the gas station is a safety system, it is found that there is only one major fire risk point in the whole unit. In addition, since the correction coefficient (*K*_*m*_) of the alarm signal, a characteristic index of dynamic monitoring of high risk, is dynamically modified for the inherent risk (*h*) of risk points, as shown in Equation 6, the dynamic correction of the inherent risk index of the construction unit is as follows:


(6) $$\begin{array}{l} H=hK_{m} \end{array}$$

where *H* is the modified value of the dynamic monitoring index of inherent risk point.

(2) Unit initial high risk As shown in Equation 7, the high-risk risk control frequency (*G*) of the unit is aggregated with the inherent risk index (*H*) of the unit to determine the initial safety risk value of the unit. The risk level is determined in Table 2.


(7) $$\begin{array}{l} R_{0}=GH \end{array}$$

**Table 2:** Classification of realistic risk levels.

**Unit realistic risk(*R*_0_ or R)**	**Unit realistic risk level**	**Risk level symbol**
*R*<48	Low risk	IV level
48≤*R*<105	General risk	III level
105≤*R*<150	Larger risk	II level
*R*≥150	Major risk	I level

where *R*_0_ is the initial safety risk value of the unit.

(3) Actual risk of system security System actual risk *R* is the result of revising the initial high risk (*R*_0_) of the modified unit by other dynamic risk indicators. Other dynamic risks do not always exist. If this index does not exist, its modified value is 1. If it does exist, modifying *R*_0_ should be done by referring to Table 1. Finally, the actual safety risk of the fire prevention system is calculated (Equation 8):


(8) $$\begin{array}{l} R={R_{0}}^{*}{k_{c}}^{*}k_{i} \end{array}$$

where *k*_*i*_ is to increase the risk correction coefficient. Among the dynamic indicators, the special period index, early warning disposal index, and natural environment index appear, which should and will increase the risk. *i* is the adjustment times of these three indicators, *i* = 1,2,3; while *kc* is to reduce the risk correction coefficient. Note, that due to the existence of a comprehensive management index, when the risk is managed in time, the dynamic risk is significantly reduced, and should be corrected.

The values of *k*_*i*_ and *k*_*c*_ are shown in Table 1. If *k*_*i*_ and *k*_*c*_ do not exist, that is, there is no dynamic index, both *k*_*i*_ and *k*_*c*_ take the value of 1.

(4) Actual risk level of the unit The risk regulated by the risk control index and dynamic risk index is regarded as the actual risk of the final fire event, as shown in Table 2. The realistic safety risk level of a gas station system is divided into I, II, III, and IV (38).

## Case study

The construction area of a city gas station is about 1,000 square meters, generally used to add fuel oil, lubricating oil, and so on. There are 5 gasoline tanks with a total of 260t, and the critical value of gasoline is 200t. The correction coefficient is 2.1, and convenience stores with other convenience measures are supported. The gas station area is equipped with 7 types of monitoring facilities, such as an electrostatic eliminator, fire alarm devices, and video surveillances, with a normal daily online monitoring system. The risk control frequency index of fire occurrence is 0.13. The gas station staff of 5 people, and the fire accident, may affect the number of surrounding residents reaching 80 people, the daily inspection found 15 hidden dangers, and an emergency plan. Based on the above examples, the risk point of a gas station fire accident is taken as the evaluation object, the system risk assessment model is applied to these examples, and the feasibility assessment analysis is carried out.

(1) Inherent risk index of fire risk points Gas stations contain tanks of diesel, gasoline and their attached facilities, which are high risk. Therefore, the corresponding relationship between the essential safety level of tanks and the risk index is shown in Table 3.

**Table 3:** The corresponding relationship between intrinsic safety level of tanks and hazard index.

**Indicator**	**Indicator description**	**Characteristic value**
Oil tank	Advanced equipment	1.10
	The tank is in good condition	1.30
	Poor quality of tank body	1.70
Affiliated facilities	Fully equipped and in good condition	1
	The warning system is in good condition	0.90
Risk index of dangerous equipment and facilities (*h_*s*_*)		1.70

According to the “oil tank” and “auxiliary facilities” indices to classify quantification, each index selects a characteristic value that is multiplied, with the solution result being a risk index of high-risk equipment and facilities characteristic value of 1.70.

Regarding the hazard factor of fire incident risk point, the material coefficient *M* is determined by the fire, explosion, toxicity, and other characteristics of dangerous goods at the event risk point, mainly characterized by the combustibility and chemical activity of the substance, and it describes the internal characteristics of the energy released by the fire, explosion and other chemical reactions of the substance. The larger the quantity of storage, the greater the possible consequences of instability. Therefore, according to the hazard classification standard stipulated by the identification of major hazard sources of hazardous chemicals, the ratio between the actual quantity of hazardous substances and the critical quantity at the fire event risk point was determined, and the *M* was measured by the sum of the ratio corrected by the correction coefficient β of the corresponding hazardous chemicals. The dangerous substance at the fire event risk point is calculated as follows:


(9) $$\begin{array}{l} M=\beta_{1}\frac{q_{1}}{Q_{1}}+\beta_{2}\frac{q_{2}}{Q_{2}}+\cdots +\beta_{n}\frac{q_{n}}{Q_{N}} \end{array}$$

where *M* is the coefficient of chemical combustibility or active characteristic substance;*q*_1_, *q*_2_, ⋯ *q*_*n*_ are the actual quantity of each dangerous substance; *Q*_1_, *Q*_2_, ⋯ *Q*_*n*_ are the critical quantity corresponding to each dangerous substance;β_1_, β_2_, ⋯ β_*n*_ are the correction coefficients corresponding to each dangerous substance.

In our case, the high-risk item is gasoline, and *M* is equal to 2.73 as determined by Equation 9.

High-risk sites are refueling areas where residents can be affected by the fire. A total of 85 people work in the refueling area due to the number of residents nearby. The corresponding relationship between the number of people that can be affected by the fire and the risk index is shown in Table 4.

**Table 4:** Assignment table of risk point exposure personnel index.

**Number of people exposed**	**Characterization value *E***
More than 100 people	9
30–99	7
10–29	5
3–9	3
0–2	1
Index of Persons exposed to dangerous places(*E*)	5

A high-risk process is composed of monitoring and monitoring systems. Considering the high impact and high-risk characteristics of fire and explosion, effective monitoring is an important part of ensuring the safety of gas stations, and the effectiveness of monitoring and monitoring system technology has an important impact on the refueling area. The case involved 7 types of monitoring facilities, and the monitoring status was intact, so *K*1 = 1 was obtained. A high-risk operation is determined by the type of operation in the refueling area, and so *K*2 is equal to 1.5.

Therefore, the inherent risk index of fire accident risk points can be obtained from Equation 3, calculated as *h* and equal to 48.73.

(2) Probability of risk point control As for the calculation of risk changes in daily management, the probability index of fire accident risk point control can be obtained from Equation 4, calculated as *G* and equal to 1.13.

(3) Systematic realistic risk assessment Risk dynamic simulation and risk evolution should be considered for a gas station. Since the online monitoring system is normal, it can be seen from Equation 5 that the correction coefficient of the alarm signal of the characteristic index of dynamic monitoring of high risk is 1. According to Equations 6 and 7, the result of dynamic correction of the inherent risk index of the calculation unit is 48.73, and the initial risk result of the unit is 55.07, belonging to general risk. According to Equation 8, the actual risk result of system safety is 71.59, belonging to a large risk and the risk level II. Compared with the actual situation, the realistic risk assessment results are more consistent with the reality.

## Discussion

### Inherent risk change law analysis

To verify the effectiveness and feasibility of the method, an inherent risk assessment was conducted for the fire accident risk points of 10 gas stations. The assessment results are shown in Table 5. Because the article takes the inherent risk of fire accident risk points as the main line, the real risk is calculated after the two corrections of the control state and dynamic risk. Because it is difficult to obtain the corrected data, that changes based on the inherent risk, most of the updated results increase the real risk data, so the risk change law can be analyzed according to the main data results of the inherent risk.

**Table 5:** Inherent risk assessment results of fire risk points in 10 gas stations.

**Serial number of 10 gas stations**	**Accident risk point**	***h_*s*_***	***M***	***E***	***K_1_***	***K_2_***	***h***
1# gas station	Fire	1.30	3	7	1.10	1.20	36.04
2# gas station	Fire	1.70	4	7	1.30	1.30	80.44
3# gas station	Fire	1.50	5	5	1.30	1.40	68.25
4# gas station	Fire	1.70	6	9	1.50	1.50	206.55
5# gas station	Fire	1.70	3	9	1.20	1.20	66.10
6# gas station	Fire	1.50	6	7	1	1.30	81.90
7# gas station	Fire	1.10	8	3	1	1.40	36.96
8# gas station	Fire	1.30	9	7	1.10	1.50	135.14
9# gas station	Fire	1.50	2	5	1.60	1.60	38.40
10# gas station	Fire	1.70	5	3	1.50	1.70	65.03

(1) The established system security risk assessment model highlights the focus on prevention and control. Mainly from “dangerous equipment and facilities, dangerous materials or energy, dangerous place, dangerous process, dangerous operation” to highlight the prevention and control role of key people, equipment, processes, sites, and other risks, that can fully control the actual situation, further explaining the rationality of the model evaluation results.

(2) Material hazard index (*M*) and site personnel exposure index (*E*) accounted for most of the assessment, that is, the higher the mass of the fuel, the higher the *M*; The greater the number of people affected by the fire explosion, the greater the *E*.

(3) The normal operation of online monitoring and monitoring facilities can effectively control the safe operation process parameters of the gas station system and reduce the inherent risk, which would otherwise increase. In addition, the more types of special operations used, the higher the potential inherent risks; Reducing the number of high-risk workers is by implementing automation is one of the effective ways to reduce the inherent risk at the gas station.

### Risk classification prevention and control

(1) Risk classification supervision According to the risk assessment results, the technical and supervisory countermeasures and suggestions for eliminating or weakening risks and hazards should be put forward, and prevention and control measures should be taken to reduce risks until the risk reduction is within the acceptable range. The risk of fire is divided into four grades from high to low: red, orange, yellow, and blue, and the “red, orange, yellow, and blue” safety supervision and management warning mechanism must be constructed and implemented. According to the risk value calculated by the risk classification model and based on as low as reasonably practicable, the risk assessment level of the supervised object is graded into four levels, namely, major risk, higher risk, general risk, and low risk. According to the scientific and reasonable “matching regulation principle,” the corresponding level of regulatory measures should be applied to the corresponding level of risk objects. For example, the supervision objects of major risk levels should be subjected to high-level regulatory measures, as shown in Table 6. Safety supervision departments at all levels should combine their regulatory power according to different risk levels, formulate scientific and reasonable law enforcement inspection plans. Concerning the law enforcement inspection frequency, the respect such as law enforcement inspection should focus on differentiation and encourage enterprises to strengthen the self-management, enhance the level of safety management, and adopt effective risk control measures and efforts to reduce risk.

**Table 6:** Matching supervision principle of risk classification and risk level.

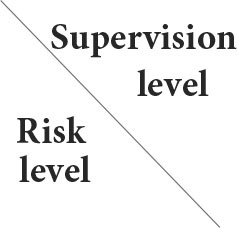	**Risk status, regulatory** **countermeasures and measures**	**Level and status of supervision**			
		**Major risk**	**Higher risk**	**General risk**	**Low risk**
I Level (major risk)	Unacceptable risk: significant regulatory measures; First-level warning: strong supervision; Comprehensive investigation, rectification.	Reasonable Acceptable	Unreasonable Unacceptable	Unreasonable Unacceptable	Unreasonable Unacceptable
II Level (higher risk)	Expected risk: large risk regulatory measures; Second-level warning: strong supervision; High-frequency inspection.	Unreasonable Acceptable	Reasonable Acceptable	Unreasonable Unacceptable	Unreasonable Unacceptable
III Level (general risk)	Limited risk acceptance: general risk regulatory measures; Third level warning: medium supervision; Local restrictions: limited checks, warning policies, etc.	Unreasonable Acceptable	Unreasonable Acceptable	Reasonable Acceptable	Unreasonable Unacceptable
IV Level (low risk)	Acceptable risk: negligible; Fourth-level warning: weakening supervision; Attention strategy: random check, etc.	Unreasonable Acceptable	Unreasonable Acceptable	Unreasonable Acceptable	Reasonable Acceptable

Red indicates that the risk result is extremely serious and may cause catastrophic consequences. The risk result is unacceptable and prevention and control measures must be taken immediately.

Orange is second, indicating that the risk result is serious, the risk result is unacceptable, and the prevention and control measures should be taken immediately.

Yellow indicates that although the risk result is not controllable, it is acceptable, and daily control needs to be strengthened.

Blue indicates that the risk result is within the control range, acceptable, and strengthen control.

(2) Implement inherent risk classification control According to the evaluation results, we should focus on high-risk processes, equipment, articles, sites, and operations, and strengthen dynamic risk control, which is conducive to prevention and control. Enterprises should implement “three simultaneous” for dangerous equipment and facilities, strictly follow the design and safety regulations, and improve the essential safety level of equipment and facilities. For inflammable materials that may lead to accidents, strict control of the parameters of high-risk materials according to relevant safety standards and design requirements is required, accompanied by daily detection and maintenance management. Enterprises should reduce personnel exposure in dangerous areas, adopt measures of “automatic personnel reduction and mechanical personnel replacement,” promote remote inspection technology, and monitor mobile personnel. Strengthening the control of the dangerous processes and improving the reliability of key monitoring dynamic data would also be essential. The employees would receive education and training on topics pertaining to production safety, production safety, and safety risk control measures.

(3) Improve the standard management level of enterprise safety Establishing hidden dangers and illegal intelligent identification system, while also strengthening hidden danger investigation and report would also be important.

(4) Strengthen dynamic risk control According to the dynamic early warning information, natural disaster, special periods, and other relevant information must be used to take timely response measures and reduce dynamic risks. Improving the real-time effectiveness of risk dynamic index data is important to avoid data distortion.

**Figure 4:**
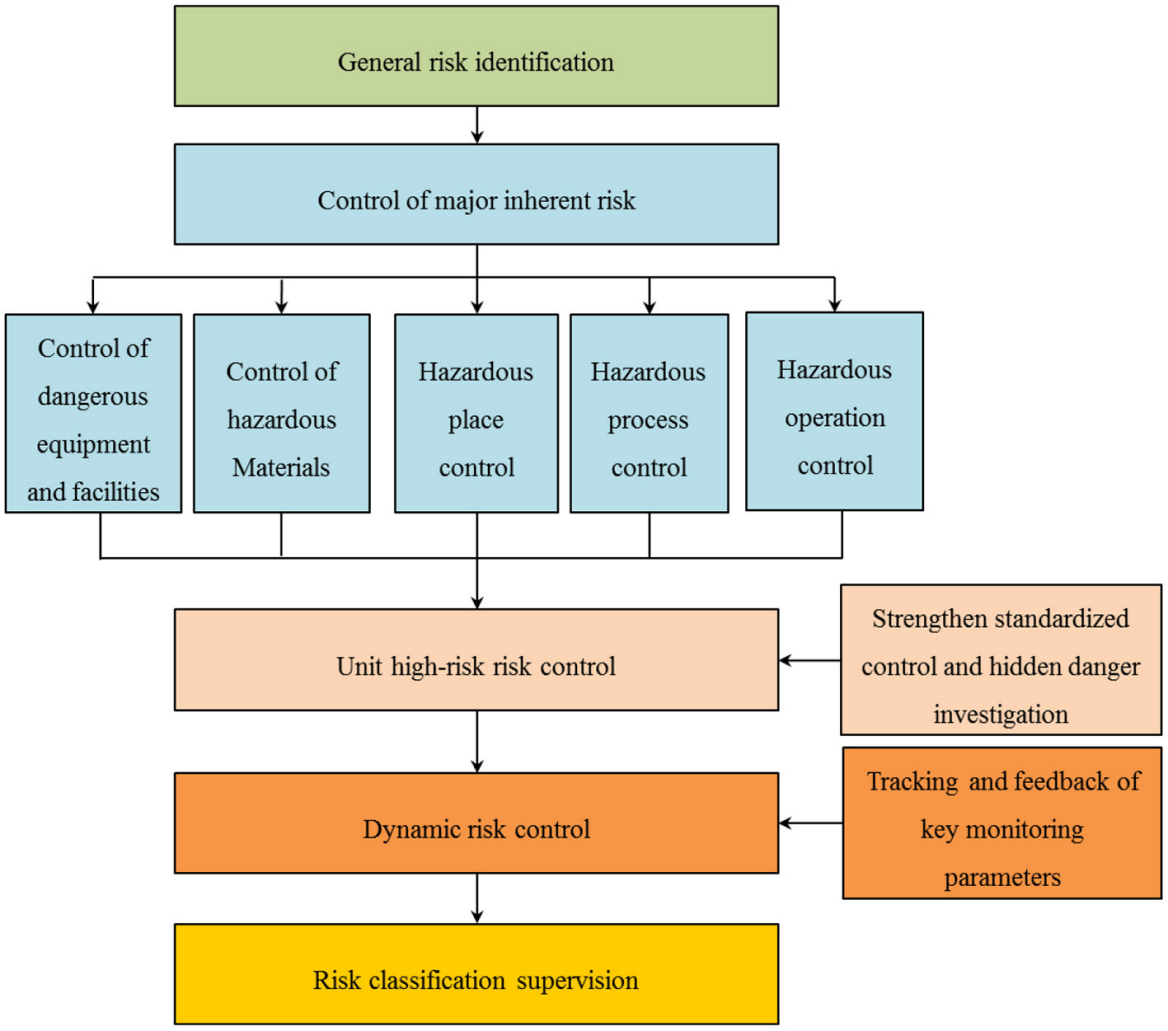
Classified management and control of security risks based on realistic risk assessment technology.

Moreover, risk classification control is implemented over five levels: general risk list identification control, major risk control, unit high-risk risk control, dynamic risk control, and risk classification supervision, as shown in Figure 4. Through targeted and advanced measures for prevention and control, improving safety prevention and control ability is possible. This further indicates that risk assessment technology is beneficial for enterprises in implementing timely and synchronous dynamic monitoring and decision-making.

### Advantages of realistic risk assessment method

Realistic risk assessment technology is developed based on the inherent attributes of the system, and the risk assessment object is divided over three stages. The first stage comprises the inherent risk, which mainly evaluates the risk of equipment, facilities, processes, materials, places, and operations, and highlights the key prevention and control parts; The second stage encompasses the unit management and control risk, which mainly assesses the risk in the management processes; The third stage is concerned with dynamic risk regulation, which mainly evaluates the impact of dynamic changes such as high-risk online monitoring, monitoring-characteristic indicators, special period indicators, and natural environment on the initial risk. The realistic risk assessment tool based on the system accident risk point is helpful to clarify the coordination between the system risk elements and the assessment objects.

The results of the realistic risk assessment methods are usually expressed by the index data obtained from statistical data or given certain data rules, and are processed and sorted in a mathematical way to obtain the risk value. This method can not only reflect the local dynamic variability of risk management and control indicators, but also facilitate the proposal of targeted risk management and control measures, further improving the accuracy of risk management prevention and control objects. This may also solve the difficult and static problem of obtaining risk analysis data in the process of complex and uncertain system management. This evaluation method may quantitatively analyze the relationship.” This may summarize the sentence in a more concise manner. Kindly check may quantitatively analyze the relationship of a given enterprise's safety status; The results of risk assessment are convenient for researchers or risk decision makers to make effective judgments and interpretations through the comparative analysis of these data.

## Conclusions

Compared to the previous simple risk assessment model, the fire accident risk point system discussed in this study is taken as the main line of risk assessment, and the transformation process of the risk points from inherent risk, to initial risk, and then to a real risk is explored, redefining the major risk assessment model and risk perception path. The static risk characterization method of the inherent attributes of dangerous equipment, facilities, materials, places, processes, and operations, in a fire safety system, is studied. Combined with the possible degree of occurrence of fire risk points during daily operations and management, the initial risk of fire risk points under local dynamic conditions is determined. With the update of a fire complex system information and abnormal information, the dynamic risk correction method is proposed again to correct the initial risk. Finally, a static risk assessment model, a local dynamic risk assessment model, and a realistic risk assessment model that can modify the initial risk are established. The system's timely risk assessment, combined with system attributes and management status, is realized, making the enterprise risk management status timely adjusted, and transparent. The shortcoming of the study is that there are other types of disasters besides fires that can occur in gas stations. The previous study was oriented to curb major accidents and focused on preventing major risks.

## Data availability statement

The raw data supporting the conclusions of this article will be made available by the authors, without undue reservation.

## Author contributions

WL: conceptualization, formal analysis, funding acquisition, investigation, methodology, software, validation, visualization, roles, writing—original draft, and writing—review and editing. XL: data curation, formal analysis, funding acquisition, methodology, project administration, resources, visualization, and writing—review and editing. XD: resources, and writing—review and editing. All authors contributed to the article and approved the submitted version.

## Funding

This research was supported by the Science and Technology Research Program of the Education Department of Hubei Province, China (Grant No. Q20212906), the High-level Cultivation Program of Huanggang Normal University, China (Grant No. 204202112304), and the Talent Initiation Program of Huanggang Normal University, China (Grant No. 2042021014).

## Conflict of interest

The authors declare that the research was conducted in the absence of any commercial or financial relationships that could be construed as a potential conflict of interest.

## Publisher's note

All claims expressed in this article are solely those of the authors and do not necessarily represent those of their affiliated organizations, or those of the publisher, the editors and the reviewers. Any product that may be evaluated in this article, or claim that may be made by its manufacturer, is not guaranteed or endorsed by the publisher.
